# The Effect of Physical Activity on Cognitive Performance in an Italian Elementary School: Insights From a Pilot Study Using Structural Equation Modeling

**DOI:** 10.3389/fphys.2019.00202

**Published:** 2019-03-05

**Authors:** Johnny Padulo, Nicola Luigi Bragazzi, Andrea De Giorgio, Zoran Grgantov, Sebastiano Prato, Luca Paolo Ardigò

**Affiliations:** ^1^Department of Psychology, eCampus University, Novedrate, Italy; ^2^Faculty of Kinesiology, University of Split, Split, Croatia; ^3^Sport Performance Laboratory, University of Split, Split, Croatia; ^4^Research Laboratory “Sport Performance Optimization”, National Center of Medicine and Sciences in Sport (CNMSS), Tunis, Tunisia; ^5^Postgraduate School of Public Health, Department of Health Sciences (DISSAL), University of Genoa, Genoa, Italy; ^6^School of Exercise and Sport Science, Department of Neurosciences, Biomedicine and Movement Sciences, University of Verona, Verona, Italy

**Keywords:** physical activity, cognitive effect, elementary school, exercise for health, structural modeling

## Abstract

When compared to the previous generations, younger generations have become sedentary on a global level. Physical activity positively contributes to human growth and development, causing, indeed, both physiological and psychological benefits. The aim of the current study was examining the relationship between physical activity and school achievement in a sample of 80 Italian elementary (viz. primary) school last year responding children (11.0 ± 0.3 years, 1.46 ± 0.09 m, 39.5 ± 7.9 kg). Such an aim was fulfilled by investigating eventual correlations between physical tests results and school marks and by disclosing eventual mutual relationships between socio-demographics, family context, lifestyle (including physical activity), and school performance information using a structural modeling approach. Children were assessed for lower/upper limbs muscle strength and running/agility performance. Pearson’s correlation between physical tests and school performance was studied. We found that agility correlated with English, Italian, mathematics, music, and sport marks, whereas jump correlated with English, mathematics, sport, and technologies marks. Sprint correlated with mathematics, sport, and technologies marks. All correlation coefficients were moderate, except for correlations between sport marks and physical tests (strong correlation). From the structural model, we found that socio-demographics and lifestyle significantly impacted on school achievement. In particular, lifestyle was found to fully moderate the impact of the family context on school achievement. Schools and households represent important settings for improving children physical and psychological-cognitive health and status, offering physical activities opportunities.

## Introduction

When compared to the previous generations, the younger ones have become sedentary on a global level, experiencing an increasingly less physically demanding life (Owen et al., 2010), and being, as such, more vulnerable to developing overweight and obesity and suffering from their long-term health consequences, like dysmetabolic disorders and tumors (Mitchell and Byun, 2014).

Work sites, time spent using digital media and virtual social contacts are creating a relevant time debt for sport and related physical activity (Corder et al., 2015), leading people to adopt sedentary behaviors (Owen et al., 2010). Physical activity, on the contrary, positively contributes to human growth and development, preparing children for the mental and physical challenges of adolescence and emerging adulthood (Prakash et al., 2015; Felfe et al., 2016). There is a huge body of evidence indicating that physical activity produces, indeed, both physiological and psychological benefits, being associated with better mental health and enhancement of brain function and cognition (Donnelly et al., 2016).

In a series of meta-analyses (Ahn and Fedewa, 2011; Jackson et al., 2016), small but significant improvements in mental health and in the domain of executive functioning (in particular, inhibitory control) were reported. These findings were confirmed by the meta-analysis performed by de Greeff et al. (2018), which documented improvements of executive functions as well as of attention. Sibley and Etnier (2003) found that even physical activity of low intensity or for a short term is related to improvements in executive and neurocognitive functioning, being the relationship not strictly dose-dependent, but both quantitative and qualitative (Pesce, 2012). Jäger et al. (2014) investigated the effects of an acute 20-min physical activity intervention on executive functions in a sample of 52 young elementary school children, aged 6–8 years old versus other 52 children assigned to a resting control condition. Authors found statistically significant cognitive effects induced by acute sports intervention.

Acute physical activity significantly improves attention. Concerning the link between physical activity and subsequent future school performance, a systematic review of the literature established a long-term relationship between the two variables under scrutiny (Singh et al., 2012). Besides the above-mentioned psychological effects, authors offered several plausible biological mechanisms underlying the reason(s) why exercise might be beneficial for cognition, including (1) increased oxygenation to the brain (Ide and Secher, 2000); (2) increased levels of neurotransmitters, including norepinephrine, epinephrine, and serotonin, associated with memory, information processing and other neuropsychological skills (Meeusen and De Meirleir, 1995); (3) release of molecules like endorphins, endogenous opioid neuropeptides, which result in stress reduction (Goldfarb and Jamurtas, 1997; Yang and Chen, 2017); and (4) increased levels of growth factors, such as brain-derived neurotropic factor (BDNF), growth hormone, and insulin growth factor 1 (IGF-1), associated with cellular development, angiogenesis, neurogenesis, and synaptogenesis (Alesi et al., 2015; De Giorgio et al., 2018).

Given these speculated processes, it is not unexpected that being involved in physical activities predicts better classroom engagement and participation. A recently published meta-analysis by de Greeff et al. (2018) quantitatively confirmed the relationship between sports and academic achievements. However, despite many researches being conducted on this topic, studies exhibit a high degree of variability and inconsistency concerning both study quality and results (Howie and Pate, 2012). The topic of the link between physical activities and school performance remains urgent and of crucial importance, given the convergence of an increasing focus on academic achievements and the decreasing physical activities opportunities in schools, worldwide (Howie and Pate, 2012).

The aim of the current study was to examine the relationship between physical activity and school achievement by 1) investigating eventual correlations between physical tests results and school marks and 2) disclosing eventual mutual relationships between socio-demographics, family context, lifestyle, and school performance information using a structural modeling approach. We used a structural model since this method enables to overcome the shortcomings that plague a cross-sectional study, showing reciprocal causal relationships among variables and having both explanatory and predictive power. Furthermore, it can estimate the multiple and interrelated dependences among variables in a single analysis (Jeon, 2015). The use of structural modeling equations and the exploration of different variables, including family context, represent the main novelties of the present study.

## Materials and Methods

### Participants

Specifically for correlational analysis sample size calculation purposes, a pilot study that included a sample of 50 children was performed *a priori* and gave a statistical power greater than 0.81. Therefore, one hundred and six voluntary children (80 responders, 11.0 ± 0.3 years, 1.46 ± 0.09 m, 39.5 ± 7.9 kg; further details in Tables 1, 2) were recruited to participate in this study. The criteria of inclusion were: (i) attending the last year of the Italian primary school. The *criteria* of exclusion were: (i) not having completed 5 years as students of the same school in which the present investigation was carried out, and (ii) suffering from neurological, cardio-vascular or orthopedic disorders that might impair their abilities to execute a battery of explosive exercises. Twenty-six children were excluded, on the basis of the first exclusion *criterion*, whereas none was excluded due to the second *criterion*.

**Table 1:** Socio-demographics and family context of the recruited sample.

Parameter	Value
**Socio-demographics**	
Body Mass Index (mean ± standard deviation)	18.45 ± 2.66
Citizenship	
Italian (n; %)	70 (87.5%)
Non Italian (n; %)	10 (12.5%)
Gender	
Female (n; %)	46 (57.5%)
Male (n; %)	34 (42.5%)
**Family context**	
(living at) Floor	
Ground floor (n; %)	32 (40.0%)
First floor (n; %)	30 (37.5%)
Second floor (n; %)	12 (15.0%)
Third floor (n; %)	6 (7.5%)
Number of family (parents + offspring) members	
Three members (n; %) Four members (n; %)	14 (17.5%) 48 (60.0%)
Five members (n; %)	18 (22.5%)
Number of family members practicing sport (median)	2
Presence of a dog (n; %)	28 (35.0%)


**Table 2:** Lifestyle and school performance of the recruited sample.

Parameter	Value
**Lifestyle (i.e., daily activities)**	
Walking the dog (n; %)	16 (57.1%)
Using the lift (n; %)	2 (2.5%)
Practicing sport (n; %)	72 (90.0%)
Number of sport disciplines practiced	
One sport (n; %)	26 (36.1%)
Two sports (n; %)	24 (33.3%)
Three sports (n; %)	8 (11.1%)
Four sports (n; %)	6 (8.3%)
Five sports (n; %)	8 (11.1%)
Practicing team sports (n; %)	32 (44.4%)
Practicing individual sports (n; %)	62 (86.1%)
Weekly hours of sport practice (mean ± standard deviation; median)	3.50 ± 3.21 (2.5)
Hours spent playing video games (mean ± standard deviation; median)	5.84 ± 6.61 (3.25)
Hours spent watching TV (mean ± standard deviation; median)	9.99 ± 7.19 (10.0)
**School performance**	
Absences from school (days, mean ± standard deviation; median)	7.93 ± 7.21 (6.0)
Italian (mean ± standard deviation; median)	7.74 ± 1.03 (7.9)
Maths (mean ± standard deviation; median)	7.63 ± 1.06 (7.6)
English (mean ± standard deviation; median)	8.03 ± 0.84 (8.2)
Sciences (mean ± standard deviation; median)	7.66 ± 0.93 (7.6)
History (mean ± standard deviation; median)	7.77 ± 0.79 (8.0)
Geography (mean ± standard deviation; median)	7.70 ± 0.82 (7.7)
Technology (mean ± standard deviation; median)	7.82 ± 0.71 (7.9)
Arts (mean ± standard deviation; median)	7.95 ± 0.81 (7.9)
Sport (mean ± standard deviation; median)	8.35 ± 0.57 (8.4)
Music (mean ± standard deviation; median)	7.99 ± 0.57 (8.2)


Before participation in this study, children and their parents were given a letter that included written information about the study and a request for consent from parents to allow their children to take part into the study. Parental informed consent was obtained after thorough explanation of study objectives, procedures, risks, and benefits. This study was conducted according to the Declaration of Helsinki. The study also conformed to the University of Split, Human Research Ethics Committee guidelines. The Human Research Ethics Committee of the University of Split approved the experimental protocol.

### Protocol

The primary outcome of the present investigation was the academic performance for different disciplines (namely, Italian language and literature, mathematics, English language and literature, sciences, history, geography, technology, arts, sport, and music).

Grades were obtained directly from the school board for each term based on examinations prepared and administered according to the ministerial guidelines. Grades were on a numeric scale and ranged from 1 to 10.

A battery of physical tests validated for children, including standing long jump and medicine ball throw to assess muscle strength for lower/upper limbs, and sprint/agility test to assess running/agility performance, was administrated (Duncan et al., 2018; Scheuer et al., 2019). Such tests (repeated after 1 week to assess reliability of the measures) were randomly administered paying particular care to minimize any potential interference with usual children schedule: more in detail, physical tests were performed at the same time of day each day (at 11.30 a.m.), in the school gym.

A standardized warm-up consisting of jogging, dynamic stretching (Chaouachi et al., 2017), and a series of increasing-intensity sprints was performed before testing, in order to better prepare the children to the execution of the physical tests. For each test, three repetitions were performed with 1–2 min of passive recovery in-between. The best performance obtained was used in our statistical analysis. For each test the time spent was 20 min.

#### Long Jump

Participants stood stationary with toes aligned with the start line, and were instructed to push off vigorously and to jump forward as far as possible. Participants were allowed to make use of a counter-movement with arms and body swing (Padulo et al., 2014). Distance jumped from the start line to position of heel at touchdown was measured in centimeters using a metal measuring tape. The test was repeated three times and the maximum distance achieved was used for further statistical analysis.

#### Free Ball

Children were asked to sit on a chair with their shoulders in contact with the chair-back. Then, they were asked to throw forward the medicine ball as far as possible for three times with 1 min of passive recovery in-between. The used medicine ball weighed 2 kg (Padulo et al., 2014). The distance from participants’ shoulders to the point of impact of the ball with the ground was measured and the longest distance was used for further analysis. The throw distance was measured by means of the measuring tape.

#### Sprint

Running ability was evaluated using a maximal 10-m sprint. Children were instructed to run as quickly as possible along a 10-m distance after a standing start. Time was automatically recorded using photocell gates (Brower Timing Systems, Salt Lake City, UT, United States; accuracy of 0.01 s) placed 0.4-m above the ground (Hammami et al., 2014). Subjects started the sprint when ready while standing just behind the first timing gate. Subjects performed two trials with at least 2 min of rest between them. The fastest run was used for further analysis.

#### Agility Test

The used agility test was 4 × 9-m shuttle run test (Kibele and Behm, 2009). Children stood behind a starting line and started the electronic clock by passing through the first timing gate. At the end of the first 9-m section, subjects were asked to step with one foot beyond a marker while reversing the running direction and sprinting back to the start where a same reversal was required. After the fourth 9-m section, participant passed through the second timing gate to stop the electronic clock. For time measurement, this test was conducted using the same equipment as in the sprint tests.

### Statistical Analysis

To quantitatively assess the relationship between physical tests and school performance, Pearson’s correlation was computed. Based on obtained *r* coefficient values, qualitative interpretation of correlation strength was carried out using the following rule of thumb proposed by Cohen (1988). Namely, values up to 0.100, between 0.100 and 0.300, and above 0.300 indicated weak, moderate, and strong relationship, respectively.

In order to properly investigate the impact of physical activity on school performance, using partial-least squares structural equation modeling analysis (PLS-SEM), a conceptual model obtained reviewing the existing scholarly literature was used. This model related to the relations between latent variables (LVs) and their corresponding manifest variables (MVs).

In particular, the conceptual model was based on 27 MVs, which have been subsequently grouped into 4 LVs (first three exogenous and fourth endogenous) named: socio-demographic variable (consisting in ethnicity/citizenship, age, gender, and body mass index or BMI), family context (consisting in the number of family members, in the number of family components practicing sport, floor of the house where family lives, and presence of a dog), lifestyle behaviors (consisting in the use of a lift, walking the dog, number of sports disciplines practiced and their type, team or individual sports, weekly hours spent practicing sport in average, and years practicing sport) and school performance.

In PLS-SEM, the model is generally described by two components referred as (1) measurement model (or construct), which relates MVs with their corresponding LVs, and (2) structural model which shows the relationship between various LVs. These relations can be pictorially shown as a path diagram, in which LVs are usually drawn with an oval shape, while rectangular shaped elements represent MVs.

The commercial software SmartPLS was used to model data concerning physical activities and school performance. A 2-step procedure (measurement model plus structural model), as recommended by Henseler et al. (2009) was adopted.

Concerning the measurement model, in order to check for reliability of the measurement model at the indicator level, the standardized outer loading for each indicator and its corresponding structure was calculated and investigated. The acceptable levels of outer loading were values higher than 0.7, as recommended by Henseler et al. (2009).

Also, the validity of the measurement model was examined by using discriminant validity verifying the Fornell-Larcker criterion, the assessment of potential cross-loadings, and the heterotrait-monotrait *ratio* statistics. Moreover, in order to examine convergent validity, several indicators were used, such as Cronbach’s alpha, Dijkstra-Henseler’s ρA, and average variance extracted (AVE). Usually, Cronbach’s alpha greater than 0.7, Dijkstra-Henseler’s ρA greater than 0.7, and AVE greater than 0.5 indicate a good convergent validity.

To evaluate the structural section of the model, coefficient of determination (*r*^2^) and path coefficient were evaluated. *r*^2^ equal to 0.19 or lower were considered weak. To assess reliability of the measures, Intra-class correlation coefficient (ICC) was calculated (Hopkins, 2000). Standardized root mean square residual (SRMR) for both saturated and estimated model was computed.

Statistical significance was computed calculating *t*-statistics for fitting indices such as d_ULS and d_G using bootstrapping with 5,000 iterations.

## Results

The main characteristics of the recruited sample are described in Table 1 (socio-demographics, and family context variables) and 2 (lifestyle and school performance parameters).

Of 106 eligible subjects, 80 fully answered the questionnaire (75.5% responding rate); 46 (57.5%) were females, whereas 34 (42.5%) were males; 70 (87.5%) were Italians, whereas only 10 (12.5%) were not Italians. Average BMI was 18.45 ± 2.66 kg/m^2^. Most families lived on ground or first floor, whereas only 7.5% of households lived on third floor. In 60% of cases, family comprised four members and, generally, two members practiced sport activities. Only 28 (35.0%) families had a dog; in 57.1% of cases, children took part in walking the dog. Interestingly, majority of children (90% of the sample) practiced at least one sport; 32 (44.4%) practiced team sports, whereas 62 (86.1%) practiced individual sports. 3.50 ± 3.21 (median 2.5) hours *per* week were spent practicing sport activities, whereas 5.84 ± 6.61 (median 3.25) and 9.99 ± 7.19 (median 10.0) hours *per* week were spent playing video games or watching TV, respectively.

**FIGURE 1:**
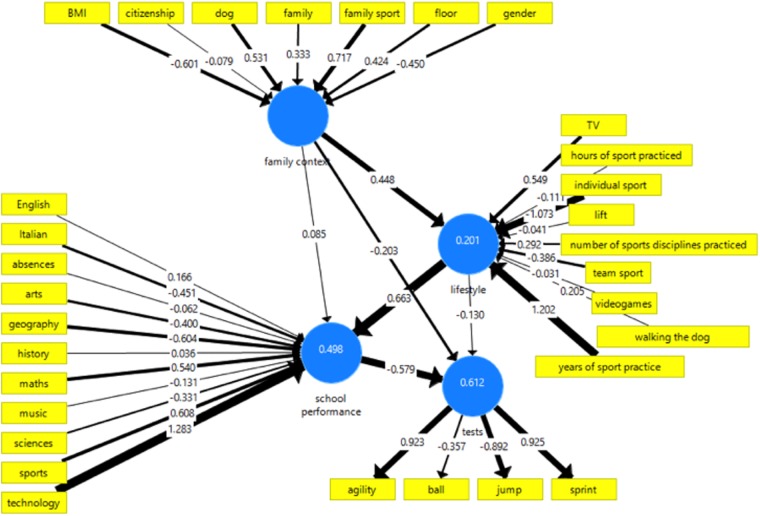
Initial full structural model investigating the impact of physical activity on school performance.

**FIGURE 2:**
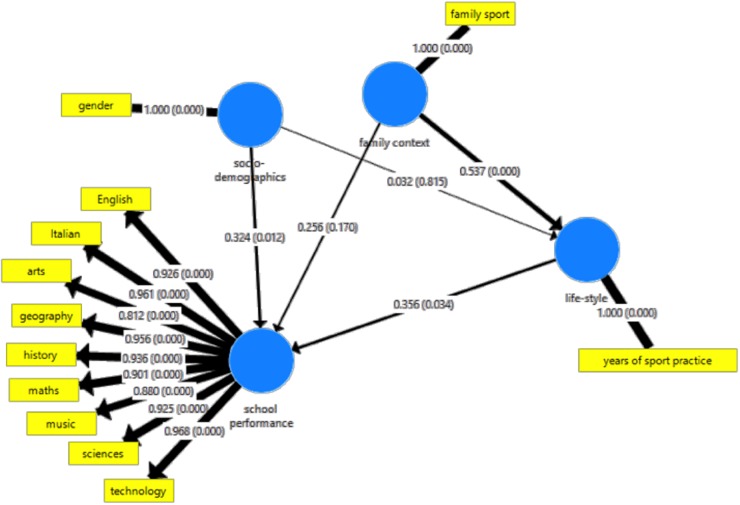
Final reduced structural model investigating the impact of physical activity on school performance.

The initial structural model is shown in Figure 1, while the final structural model obtained with 2-step procedure is depicted in Figure 2.

Fitting parameters (shown in Table 3) indicated a good fit of the model, with SRMR 0.046 and 0.049 for saturated and estimated models, respectively (being lower than the cut-off value of 0.080–0.100). d_ULS and d_G computed from bootstrapping were not significant, as expected. *r*^2^ of lifestyle and school performance variables resulted in 0.288 (adjusted 0.250) and 0.394 (adjusted 0.343), respectively, well above the cut-off of 0.190.

**Table 3:** Fitting parameters of estimated structural model (bootstrapping has been performed with 5,000 iterations).

Fitting parameter		Saturated model	Cut-off value	Estimated model	Cut-off value	
SRMR		0.046	Should be <0.080–0.100	0.049	Should be <0.0800–0.100	

**Fitting parameter (bootstrapping)**		**Value**	***t* statistics**	***P*-values**	**Cut-off value**	

d_ULS		0.186	0.493	0.622	Should be ns	
d_G		0.879	0.989	0.323	Should be ns	

**Latent variable**	***r*^2^**	***t* statistics**	***P*-values**	***r*^2^ adjusted**	***t* Statistics**	***P-*values**

Lifestyle	0.288	2.454	0.014	0.250	2.017	0.044
School performance	0.394	3.170	0.002	0.343	2.551	0.011


*SRMR, standardized root mean residual.*

Table 4 reports averages scores for agility test, free ball, long jump, and sprint, together with their ICCs, showing a good reliability of the administered physical tests.

**Table 4:** Average scores of the different physical tests and their intra-class correlation coefficients (ICC) with their 95% confidence interval (CI).

Test	Mean		Standard Deviation	
Agility test (s)	21.16		2.16	
Free ball (m)	3.96		0.63	
Long jump (m)	1.89		0.44	
Sprint (s)	2.59		0.27	

**ICC**				

**Test**	**ICC for single measures and 95% CI**		**ICC for average measures and 95% CI**	

Agility test	0.8687	0.7637–0.9290	0.9298	0.8660–0.9632
Free ball	0.7300	0.5413–0.8487	0.8439	0.7024–0.9182
Long jump	0.8469	0.7271–0.9167	0.9171	0.8420–0.9565
Sprint	0.7370	0.5519–0.8529	0.8486	0.7112–0.9206


Table 5 reports correlation coefficients between physical tests and school performance. Agility correlated with English, Italian, mathematics, music, and sports marks, whereas jump correlated with English, mathematics, sports, and technologies marks. Sprint correlated with mathematics, sports, and technologies marks. All correlation coefficients were moderate, except for correlations between sports marks and physical tests (strong correlation).

**Table 5:** Correlational analysis between physical tests and school achievements.

Pearson’s correlation	Agility	Free ball	Jump	Sprint
Arts	-0.084	-0.039	0.066	0.115
	0.6082	0.8089	0.6880	0.4815
Geography	-0.275	0.069	0.309	-0.249
	0.0863	0.6701	0.0520	0.1214
English	-0.400*	0.012	0.353*	-0.270
	0.0105	0.9415	0.0253	0.0915
Italian	-0.337*	-0.003	0.290	-0.176
	0.0333	0.9876	0.0693	0.2772
Maths	-0.423*	0.080	0.360*	-0.314*
	0.0065	0.6232	0.0226	0.0481
Music	-0.315*	0.125	0.295	-0.116
	0.0479	0.4423	0.0642	0.4769
Sciences	-0.203	-0.054	0.218	-0.182
	0.2091	0.7397	0.1762	0.2598
Sport	-0.622*	0.072	0.572*	-0.608*
	<0.0001	0.6584	0.0001	<0.0001
History	-0.143	0.005	0.144	-0.061
	0.3797	0.9738	0.3759	0.7066
Technologies	-0.381*	0.124	0.375*	-0.313*
	0.0154	0.4472	0.0171	0.0494


*^∗^statistically significant.*

Socio-demographics and life-style significantly impacted on school performance. In particular, lifestyle was found to fully moderate the impact of family context on school performance, as shown in Figure 2.

More in detail (Table 6), path coefficient between family context (family members practicing sport activities) and lifestyle context (number of years spent by child practicing sport activities) was 0.537 (*P* = 0.000), whereas the path coefficient between lifestyle context and school performance was 0.356 (*P* = 0.034), with the path coefficient between family context and school performance yielding a value of 0.256 (*P* = 0.170, not statistically significant). Path coefficient between socio-demographic variables and school performance resulted in 0.324 (*P* = 0.012), whereas path coefficient between socio-demographic variables and lifestyle context was 0.032 (*P* = 0.815, not statistically significant).

**Table 6:** Path coefficients of the estimated structural model.

Path	Path coefficient	*t* statistics	*P*-values
Family context → lifestyle	0.537	4.469	0.000
Family context → school performance	0.256	1.366	0.172
Lifestyle → school performance	0.356	2.138	0.033
Socio-demographics → lifestyle	0.032	0.231	0.817
Socio-demographics → school performance	0.324	2.453	0.014


## Discussion

Improving school performance is an appropriate goal for educational policy-makers, as improving physical activity is an onus for health promotion and sports authorities. So, determining the factors that most affect the relationship between physical activity and school performance is really important for schools, sports, and health-care workers.

Our findings that gender, years of sport practice, and family context have a positive impact on academic achievements are in line with extant literature, even though we found that 90% of our sample practiced at least one sport, which is a percentage higher than those computed by other scholars. For instance, Fox et al. (2010), drawing data from the “Project EAT” (Eating Among Teens), an extensive survey of 4,746 middle and high school students, reported participation on at least 1 sports team in the past 12 months by 53–71% of all participants.

A growing number of articles has shown strong relationships among physical activity, cognitive functions, and academic achievements in children (Hillman et al., 2008; Vazou and Skrade, 2017). As aforementioned, under “physical activity” locution are intended both quantitative (e.g., intensity and duration) and qualitative (e.g., coordinative demands and cognitive engagement) characteristics of activities. Quantitative activities lead to enhance, for example, cardiovascular fitness, whereas qualitative activities can improve, for example, motor skills (Diamond, 2015). Without being exhaustive, several studies examined the relationships between physical activity or exercise, and cognitive performance, and various results were found (Trudeau and Shephard, 2008).

According to Landry and Driscoll (2012), exercise improves health, as well as psychological well-being, cognition, and school performance. Green (2016) studied whether healthy lifestyle factors impact on academic performance among university students and found that healthy lifestyle (being physically active, no smoker, no-binge drinker, and healthy diet) increases first-year students’ performance. Effect of physical education and activity levels on academic achievement in school-age children in different categories such as perceptual skills, intelligence quotient, achievement, verbal tests, mathematic tests, memory, and readiness was showed by Ahn and Fedewa (2011), Carlson et al. (2008), Coe et al. (2006), de Greeff et al. (2018), Negi et al. (2016), and Sibley and Etnier (2003).

With regard to qualitative and/or quantitative characteristics of the physical activities, Beck et al. (2016) have demonstrated that gross motor activity integrated into math lessons could improve children mathematical performance. Authors also investigated fine motor skills, showing that these abilities are not able to improve math performances. Children studied were 165 and were tested before, immediately after, and 8 weeks after an intervention lasted 6 weeks. Beck et al. (2016) highlighted that improvement was significantly greater after gross motor than fine motor activities and that physical activities exhibited a reduced effect 8 weeks after the intervention. Furthermore, increasing the level of engagement in physical activity in childhood can also improve scores in memory, attention, and problem-solving skills. For example, Davis et al. (2011) showed in overweight and obese children that after-school physical activity intervention through vigorous intensity was able to improve executive function scores. Authors highlighted significant improving of working memory.

Palmer et al. (2013) demonstrated that physical activity intervention leads to beneficial effect of sustained attention. It is also known that physical activity can lead to weight loss. This achievement is also very important, because, as it has been discussed in a recent review by Veronese et al. (2017), weight loss was associated with improvements in cognitive function among overweight and obese people. It is worth to mention that Schmidt et al. (2016) highlighted that effects of exercise are independent from its intensity and are able to increase executive functions after 6 weeks of physical activity intervention.

Regarding adulthood, a Saudi Arabian study by Al-Drees et al. (2016) reported a positive association between physical activity habits among medical students and high academic achievement.

Motor activity effects on brain are also studied. For example, greater gray matter hippocampal volumes but lower gray matter thickness in the frontal cortex are demonstrated after higher-fit activities in preadolescent children (Chaddock et al., 2010; Chaddock-Heyman et al., 2014, 2015). It was also shown that these brain modifications lead to superior cognitive and academic performance. In particular, physical exercise can cause a release of several biomarkers such as BDNF, nerve growth factor (NGF), and IGF-1 that have a positive impact on brain structures (Skriver et al., 2014).

These findings are also confirmed by several animal studies in which it was shown that neurotrophins influences neuroplastic processes related, also but not only, to the hippocampus and, then, to memory formation (Cotman et al., 2007). Interestingly, De Giorgio (2017) and Granato and De Giorgio (2014) discussed the role of motor activity in subjects with intellectual disabilities, showing that in an experimental model of intellectual disability the motor activity is able to activate neurotropic factors, resulting into a partial repair of the neural damage.

Finally, using data from the “National Educational Longitudinal Survey”, Eitle (2005) investigated whether there are gender and race differences in the effects of participation in a variety of sports on achievement in science, mathematics, reading, and history and found that achievement benefits of playing team sports and individual sports appear to be more relevant for white female participants than for others.

The main strength of our study lies in the fact that we exploited structural modeling and, as such, this allows drawing causal conclusions from the data. On the other hand, the major shortcoming is that more running/agility performance measures would be needed to get a complete picture of children capabilities. Furthermore, future studies should replicate our findings utilizing larger sample sizes to consequently achieve greater statistical powers.

## Conclusion

Schools and households represent important settings for improving children physical and psychological-cognitive health and status, offering physical activities opportunities (Álvarez-Bueno et al., 2016, 2017).

In our investigation, we found that agility correlated with English, Italian, mathematics, music, and sport marks, whereas jump correlated with English, mathematics, sport, and technologies marks, and sprint correlated with mathematics, sport, and technologies marks. All correlations were moderate, except for correlations between sports marks and physical tests (strong correlation). From the structural model, we found that socio-demographics and lifestyle significantly impacted on school achievement: lifestyle was found to fully moderate the impact of the family context on school achievement.

Our predictive model can be useful for different stakeholders, including educational policy-makers and workers in the field of health promotion, sport, and education.

## Author Contributions

All authors listed have made a substantial, direct and intellectual contribution to the work, and approved it for publication.

## Conflict of Interest Statement

The authors declare that the research was conducted in the absence of any commercial or financial relationships that could be construed as a potential conflict of interest.
